# Fetal head station during artificial rupture of membranes: a large retrospective cohort study

**DOI:** 10.3389/fmed.2025.1612947

**Published:** 2025-10-02

**Authors:** Yishai Sompolinsky, Joshua Guedalia, Naama Vilk-Ayalon, Sarah M. Cohen, Shirley Greenbaum, Doron Kabiri, Simcha Yagel, Michal Lipschuetz

**Affiliations:** ^1^Obstetrics & Gynecology Division, Hadassah-Hebrew University Medical Center, Jerusalem, Israel; ^2^Henrietta Szold Hadassah Hebrew University School of Nursing in the Faculty of Medicine, Jerusalem, Israel

**Keywords:** obstetrics, personalized medicine, prediction, artificial rupture of membranes, fetal head station, vaginal delivery

## Abstract

**Introduction:**

Artificial rupture of membranes (AROM) is a common intervention during delivery, usually done in order to expedite delivery. Studies to determine optimal timing of AROM according to cervical dilation were inconclusive. However, other important factors, which are known to be associated with timing of delivery were ignored. One of these factors is fetal head station (FHS). We sought to investigate the association between FHS during AROM and time to delivery and other obstetrical outcomes.

**Material and methods:**

A retrospective cohort study encompassing data from labors during a 12-year period were analyzed. All cases of singleton, term pregnancy with documented AROM time were included. The study population was stratified by parity.

**Results:**

This study cohort included 45,898 singleton, term vaginal delivery parturients with time stamp at time of AROM and delivery. Stratification by parity yielded 11,947 primiparas (26%) and 33,951 multiparas (74%). Across all sub-cohorts, as fetal head station decreased at AROM the duration from ROM to delivery was shorter. This trend seems to be stronger for multiparas than primiparas. Rates of cesarean delivery, postpartum hemorrhage, neonatal intensive care unit admission, and low 5-min Apgar scores were also negatively associated with decrease in fetal head station at AROM across all cervical dilations.

**Conclusion:**

Lower fetal head station at AROM is associated with shorter time to delivery as well as lower rates of cesarean delivery, postpartum hemorrhage, NICU admission, and 5-min Apgar ≤ 7. Fetal head station should be considered alongside cervical dilation during AROM. Our findings underscore the necessity for personalized timing of AROM, especially in multiparous women, to enhance maternal and neonatal health outcomes.

## Introduction

Artificial rupture of membranes (AROM) is a common obstetric practice, usually performed to induce, augment, or otherwise expedite delivery, with the intent to shorten the duration of labor by increasing uterine activity and allowing the fetal head to apply direct pressure on the cervix.

Several studies have attempted to ascertain the optimal timing for AROM during induction of labor. A meta-analysis by De vivo et al. (1) combining 1,273 women from 4 different randomized controlled trials (RCTs), compared early vs. delayed AROM after cervical ripening and showed that early AROM was associated with a mean reduction of 5 h in the overall interval from initiation of induction to delivery. However, only one of the four RCTs (2) compared early AROM, defined as AROM before 4 cm of cervical dilation, to late AROM, defined as AROM after arriving at 4 cm of cervical dilation. In two other studies (3, 4), early AROM was compared to spontaneous rupture of membranes (SROM) and one study (5) defined late AROM after oxytocin was administered and either regular contractions or changes in cervical dilation were noted. The heterogenicity amongst these studies affects the generalizability these results.

In the past, labor augmentation of was routinely performed to shorten labor duration, following protocols pioneered by the work of O'Driscoll et al. (6), which showed lower rates of prolonged labor but were associated with complications such as increased rates of abnormal fetal heart tracings, neonatal asphyxia, and postpartum hemorrhage (7). Consequently, professional organizations such as the World Health Organization (WHO), the American College of Obstetricians and Gynecologists (ACOG) and the National Institutes for Health and Care Excellence (NICE) all issued guidelines restricting routine labor augmentation (8–10).

Early studies showed AROM to be associated with increased rates of non-reassuring fetal heart tracing and Cesarean deliveries (CD) (11, 12). More recent studies (2) demonstrated AROM to be a safe procedure with lower rates of labor dystocia and CD (13). Smyth et al. (14) in their Cochrane review did not find evidence supporting the hypothesis that AROM expedited spontaneous labor. A recent systematic review summarizing studies published between 2019 and 2024 reported that AROM shortens labor without increasing adverse outcomes (15). Similarly, a randomized trial demonstrated that amniotomy performed at 5 cm, consistent with the World Health Organization definition of the active phase of labor, shortened labor duration by ~50 min without increasing adverse maternal or neonatal outcomes (16).

The debate persists regarding both the efficacy of AROM and its optimal timing, particularly concerning early AROM and its potential benefits. Early AROM is typically defined as that performed before 4 cm of cervical dilation is reached. However, this arbitrary categorization of early vs. late AROM fails to take other routine labor assessment parameters into account, particularly fetal head station (FHS). Consideration of FHS in addition to cervical dilation might aid in optimizing AROM timing. Interestingly, although cervical effacement and FHS are part of the Bishop scoring system (17), which is used for decision making regarding the need for cervical ripening during induction of labor, these variables were overlooked in the studies addressing timing of AROM (18).

FHS has long been identified as an important predictor of labor outcomes and timing (19–21), as well as successful instrumental deliveries. However, most studies regarding FHS assessment focus on the second stage of labor.

The association between FHS and time to delivery has long been established (22, 23). Hamilton et al. (24) showed that a multifactorial model combining cervical dilation with FHS descent during the first stage of labor is more accurate in defining normal and abnormal labor progress. However, timing of AROM according to FHS has not been studied.

The aim of this study was to stratify, in a large cohort of births, the obstetrical outcomes for every cervical dilation/head station combination present at the time of AROM, in order to emphasize the importance of FHS position in the timing of AROM.

## Materials and methods

### Patients

This is an observational, retrospective electronic health records-based study performed on data retrieved from two campuses of a tertiary care center between 2003 and 2015. Data were gathered on parturients with singleton, term (≥ 37 weeks of gestation) live births.

Our institutional ethics review board reviewed and approved the study (0632-15-HMO). Given that all records were anonymized, the IRB exempted the need for informed consent for this retrospective study.

### Data collection

We gathered obstetric background and outcome data, including maternal demographic parameters (i.e., maternal age, parity, gestational age, mode of delivery), and neonatal outcomes (birthweight, 5-min Apgar score, NICU admission). The core outcome sets included a computed variable for duration of labor from AROM to delivery, which was extracted from time stamps of AROM and delivery. Researchers extracting and analyzing data were not involved in patient care; the ward staff who recorded data in real time at the point of care were not aware of the study.

FHS was recorded from −3 to +3 in our medical center.

Since primiparas differ substantially in timing of labor and obstetrical outcomes, the cohort was divided by parity to primiparas and multiparas and the groups were analyzed separately.

The primary outcome was the duration of labor from AROM to delivery, comparing each cervical dilation/FHS combination recorded at the time of AROM. A sub-cohort of patients achieving vaginal delivery was identified to minimize the possible effects of first stage CDs on timing of delivery.

Secondary outcomes included rates of CDs, instrumental vaginal deliveries, and other obstetrical complications: postpartum hemorrhage (PPH, defined as estimated blood loss of more than 500 ml and 1,000 ml for vaginal and CD, respectively), neonatal intensive care unit (NICU) admission and 5-min Apgar score ≤ 7.

### Statistical analysis

Statistical analysis was performed using Python 3.7.3 and IBM SPSS 29 for Windows (IBM Corp., Armonk, NY, USA). Dichotomous variables were compared with the chi-square test or Fisher exact test in cases of small numbers, as appropriate; the Mann–Whitney *U* test was applied to analyze differences in continuous variables. For comparing the sum ranks between the duration of labor from AROM for each station we performed Dunn's multiple comparisons test.

## Results

This study cohort included 62,657 singleton, term vaginal delivery parturients; among whom 45,898 underwent AROM with documented timestamps at time of ROM and delivery. Of these, 11,947 (26.0%) were primiparas and 33,951 (74.0%) were multiparas. Table 1 summarizes the demographic and obstetric background parameters and main maternal and neonatal outcomes for parturients who underwent AROM.

**Table 1:** Obstetric parameters of parturients with artificial rupture of membranes (*n* = 45,898).

**Parameter**	**Primiparas**	**Multiparas**
*n*	**11,947 (26.0)**	**33,951 (74.0)**
Maternal age	26 (22–29)	31 (27–35)
Gestation week	40 (39–40)	40 (39–40)
Induction	2,309 (19.3)	5,005 (14.7)
**Mode of delivery**		
Normal vaginal delivery	8,549 (71.6)	31,872 (93.9)
Assisted vaginal delivery	2,269 (19.0)	1,263 (3.7)
Unplanned cesarean delivery	1,129 (9.5)	816 (2.4)
Female neonatal	5,784 (48.4)	16,076 (47.4)
Neonatal birthweight (gram)	3,235 (2,990–3,516)	3,348 (3,078–3,623)
Neonatal head circumference^*^	34.5 (33.5–35.1)	34.5 (33.6–35.2)
Postpartum hemorrhage	717 (6.0)	1,440 (4.2)
Apgar at 5 min ≤ 7	92 (0.8)	127 (0.4)
NICU	84 (0.7)	132 (0.4)
Duration from 3 cm dilation to delivery in minutes	553 (396–753)	341 (215–504)
Duration from AROM to delivery in minutes	372 (216–583)	163 (61–325)

Data are *n* (%) or median (Inter-Quartile-Range).

^*^Neonatal head circumference is available in the database for 4,387 primiparas and 14,194 multiparas.

**Figure 1:**
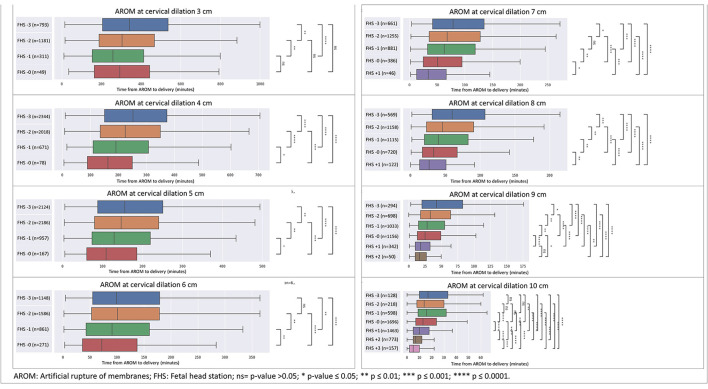
Distribution of time from AROM to birth in minutes by dilation and stratified by fetal head station at time of AROM in multiparas. The figure displays the time in minutes from AROM to delivery, based on cervical dilation and fetal head station at the time of AROM. The x-axis represents the time in minutes, while the y-axis denotes the cervical dilation stages (3–10 cm) and the corresponding fetal head stations (−3 to +3). The box plot centralizes on the median time with the box representing the interquartile range (IQR), whereas the whiskers extend to the furthest data point within 1.5 times the IQR from the box. AROM, artificial rupture of membranes; FHS, fetal head station; ns = *p* > 0.05; *p ≤ 0.05; **p ≤ 0.01; ***p ≤ 0.001; ****p ≤ 0.0001.

**Figure 2:**
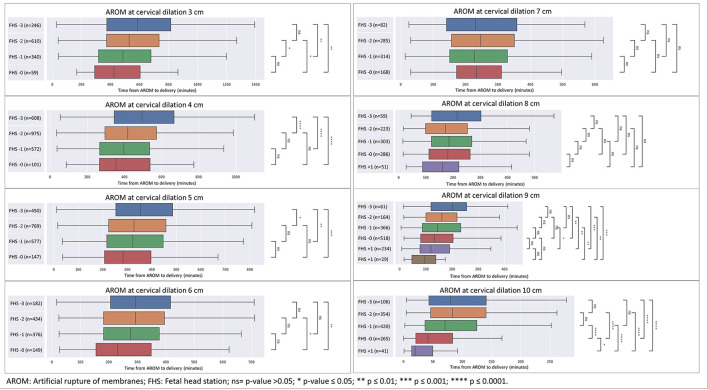
Distribution of time from AROM to birth in minutes by dilation and stratified by fetal head station at time of AROM in primiparas. The figure displays the time in minutes from AROM to delivery, based on cervical dilation and fetal head station at the time of AROM. The x-axis represents the time in minutes, while the y-axis denotes the cervical dilation stages (3–10 cm) and the corresponding fetal head stations (−3 to +3). The box plot centralizes on the median time with the box representing the interquartile range (IQR), whereas the whiskers extend to the furthest data point within 1.5 times the IQR from the box. AROM, artificial rupture of membranes; FHS, fetal head station; ns = *p* > 0.05; *p ≤ 0.05; **p ≤ 0.01; ***p ≤ 0.001; ****p ≤ 0.0001.

**Figure 3:**
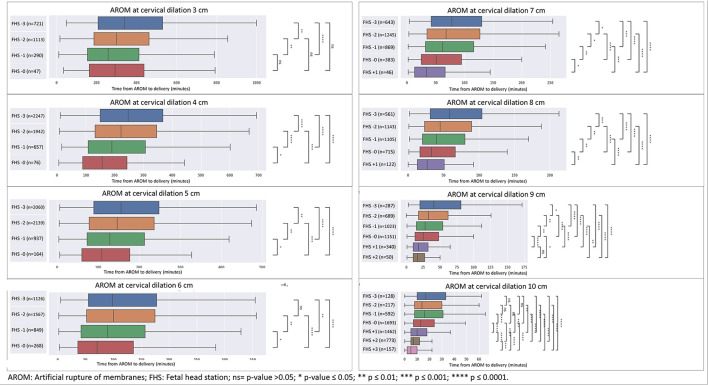
Sensitivity analysis for cohort excluding CDs—distribution of time from AROM to birth in minutes by dilation and stratified by FHS at time of AROM in primiparas. The figure displays the time in minutes from AROM to delivery, based on cervical dilation and fetal head station at the time of AROM in primiparas, excluding patients who underwent unplanned CD. The x-axis represents the time in minutes, while the y-axis denotes the cervical dilation stages (3–10 cm) and the corresponding fetal head stations (−3 to +3). The box plot centralizes on the median time with the box representing the interquartile range (IQR), whereas the whiskers extend to the furthest data point within 1.5 times the IQR from the box. AROM, artificial rupture of membranes; FHS, fetal head station; ns = *p* > 0.05; *p ≤ 0.05; **p ≤ 0.01; ***p ≤ 0.001; ****p ≤ 0.0001.

**Figure 4:**
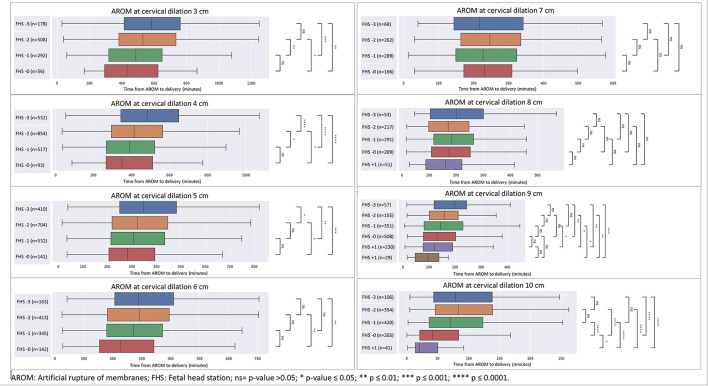
Sensitivity analysis for cohort excluding CDs—distribution of time from AROM to birth in minutes by dilation and stratified by FHS at time of AROM in multiparas. The figure displays the time in minutes from AROM to delivery, based on cervical dilation and fetal head station at the time of AROM in multiparas, excluding patients who underwent unplanned CD. The x-axis represents the time in minutes, while the y-axis denotes the cervical dilation stages (3–10 cm) and the corresponding fetal head stations (−3 to +3). The box plot centralizes on the median time with the box representing the interquartile range (IQR), whereas the whiskers extend to the furthest data point within 1.5 times the IQR from the box. AROM, artificial rupture of membranes; FHS, fetal head station; ns = *p* > 0.05; *p ≤ 0.05; **p ≤ 0.01; ***p ≤ 0.001; ****p ≤ 0.0001.

As seen in Figures 1, 2, for each cervical dilation, an advanced FHS at time of AROM was associated with shorter time to delivery interval. This statistically significant association was seen from cervical dilation at time of AROM of 3 cm to 10 cm in multiparas (Figure 1). For primiparas (Figure 2), this association was statistically significant in cervical dilation of 3–6 cm and then 9–10 cm. These results were similar in a sub-cohort of women who achieved vaginal delivery, as seen in Figures 3, 4.

Secondary outcomes—CD rates, postpartum hemorrhage, 5-min Apgar score ≤ 7 and NICU admissions were also negatively associated with advancement in FHS at AROM.

**Figure 5:**
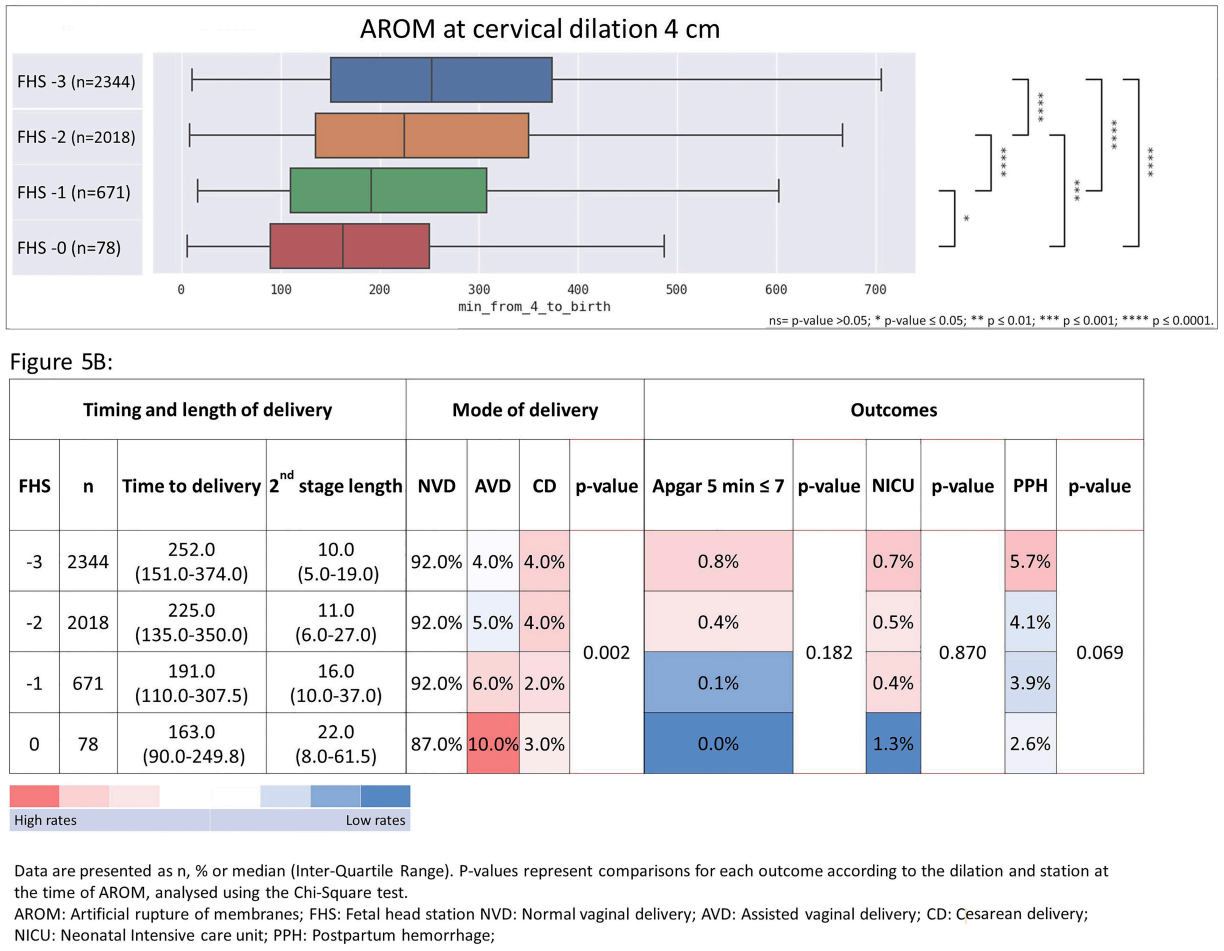
Time from AROM at cervical dilation of 4 cm to birth, stratified by FHS at time of AROM with associated heat-map summarizing timing and length of delivery, mode of delivery, and maternal and neonatal outcomes in multiparas. The figure shows the time from AROM to birth and associated outcomes, based on a cervical dilation of 4 cm, stratified by FHS in multiparas. **(A)** median delivery times with associated ranges; **(B)** the frequencies and median durations of delivery, categorized by delivery mode and delivery outcomes. Data are displayed as *n*%, or median with interquartile range (IQR). *p*-values from the Chi-Square test highlight outcome comparisons by dilation and FHS. Data are presented as *n*, % or median (Inter-Quartile Range). *P*-values represent comparisons for each outcome according to the dilation and station at the time of AROM, analyzed using the Chi-Square test. AROM, artificial rupture of membranes; FHS, fetal head station; NVD, normal vaginal delivery; AVD, Assisted vaginal delivery; CD, cesarean delivery; NICU, neonatal Intensive care unit; PPH, postpartum hemorrhage. *p ≤ 0.05; ***p ≤ 0.001; ****p ≤ 0.0001.

**Figure 6:**
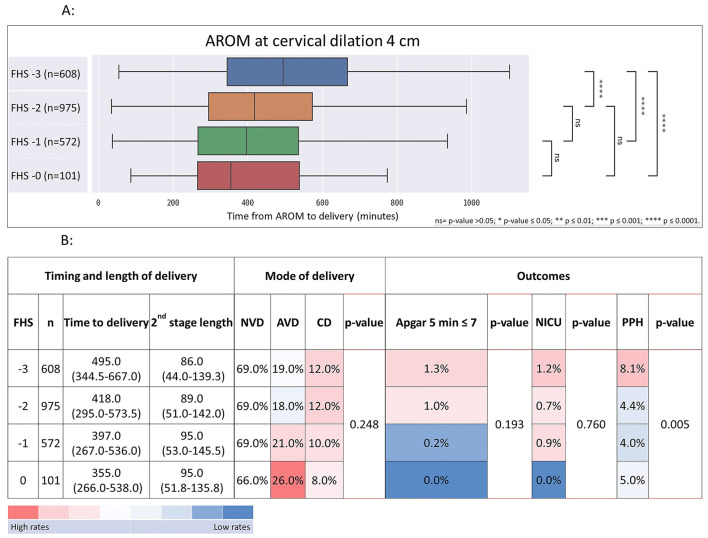
Time from AROM at cervical dilation of 4 cm to birth, stratified by FHS at time of AROM with associated heat-map summarizing timing and length of delivery, mode of delivery, and maternal and neonatal outcomes in primiparas. The figure shows the time from AROM to birth and associated outcomes, based on a cervical dilation of 4 cm, stratified by FHS in primiparas. **(A)** median delivery times with associated ranges; **(B)** the frequencies and median durations of delivery, categorized by delivery mode and delivery outcomes. Data are displayed as *n*%, or median with interquartile range (IQR). *p*-values from the Chi-Square test highlight outcome comparisons by dilation and FHS. Data are presented as *n*, % or median (Inter-Quartile Range). *P*-values represent comparisons for each outcome according to the dilation and station at the time of AROM, analyzed using the Chi-Square test. AROM, artificial rupture of membranes; FHS, fetal head station; NVD, normal vaginal delivery; AVD, Assisted vaginal delivery; CD, cesarean delivery; NICU, neonatal Intensive care unit; PPH, postpartum hemorrhage. ns = *p* > 0.05; ****p ≤ 0.0001.

Illustrating this association, we provide a breakdown of the duration from AROM at cervical dilation of 4 cm, delineated by subdivisions of FHS within this dilation and stratified by multiparas and primiparas, as shown in Figure 5 (multiparas) and Figure 6 (primiparas).

For primiparas at 4 cm of dilation (Figure 6), the time from AROM to delivery decreased with FHS advancement, while CD rates showed a declining trend. Rates of low 5-min Apgar scores and NICU admissions decreased, while postpartum hemorrhage rates significantly decreased with advancing FHS. The median time from AROM to delivery was 495 min (interquartile range (IQR) 344–667) when FHS was at −3; 418 min (IQR 295–573) when FHS was at −2, 397 min (IQR 267–536) when FHS was at −1 and 355 min (IQR 266–538) when FHS was at 0. CD rates in this group declined gradually from 12% when FHS was at −3% to 8% when FHS was at 0 (*p* = 0.248). Rates of low ( ≤ 7) 5-min Apgar scores decreased in a dose response manner as well, from 1.3% for FHS −3% to 0.0% at FHS 0 (*p* = 0.193). NICU admission rates decreased from 1.2% at FHS −3 to 0.0% at FHS 0 (*p* = 0.760). PPH rates were 8.1% when FHS was −3 and decreased significantly to 4.2%, 4.1% and 5.0% for FHS of −2, −1 and 0, respectively (*p* = 0.005).

Table 2 depicts a full heat-map table for each cervical dilation in primiparas.

**Table 2:** Heatmap of delivery outcomes and timing from AROM to delivery, stratified by cervical dilation and FHS in primiparas.

**Timing and length of delivery**					**Mode of delivery**				**Outcomes**					
**Dilation**	**Head station**	***n***	**Time AROM to delivery**	**Second stage length**	**NVD**	**AVD**	**CD**	***p*** **-value**	**Apgar 5 min** ≤ **7**	***p*** **-value**	**NICU**	***p*** **-value**	**PPH**	***p*** **-value**
3	−3	246	586.0 (376.0–816.3)	87.0 (38.0–140.0)	51%	21%	28%	<0.001	0.8%	<0.001	2.0%	0.676	9.8%	0.038
	−2	610	529.5 (374.3–736.0)	100.0 (55.0–157.5)	60%	23%	17%		1.3%		0.8%		6.1%	
	−1	340	487.0 (318.0–680.8)	98.0 (48.8–152.0)	65%	21%	14%		1.2%		1.2%		5.0%	
	0	59	424.0 (291.0–605.5)	84.0 (55.5–127.0)	69%	25%	5%		0.0%		1.7%		0.0%	
4	−3	608	495.0 (344.5–667.0)	86.0 (44.0–139.3)	69%	19%	12%	0.248	1.3%	0.193	1.2%	0.760	8.1%	0.005
	−2	975	418.0 (295.0–573.5)	89.0 (51.0–142.0)	69%	18%	12%		1.0%		0.7%		4.4%	
	−1	572	397.0 (267.0–536.0)	95.0 (53.0–145.5)	69%	21%	10%		0.2%		0.9%		4.0%	
	0	101	355.0 (266.0–538.0)	95.0 (51.8–135.8)	66%	26%	8%		0.0%		0.0%		5.0%	
5	−3	450	353.5 (253.0–485.0)	88.0 (44.5–138.5)	71%	20%	9%	0.539	0.7%	0.986	0.2%	0.889	12.0%	<0.001
	−2	769	327.0 (224.0–458.0)	85.0 (41.0–137.0)	74%	17%	8%		0.7%		0.4%		8.1%	
	−1	577	323.0 (217.0–447.0)	95.0 (50.0–148.0)	73%	19%	8%		0.9%		0.2%		4.2%	
	0	147	283.0 (207.5–396.5)	90.0 (38.3–144.5)	77%	19%	4%		0.7%		0.0%		4.1%	
6	−3	182	293.0 (206.3–416.8)	89.0 (52.5–141.0)	68%	21%	10%	0.038	1.1%	0.946	0.0%	0.002	12.1%	<0.001
	−2	434	294.0 (182.0–395.8)	86.0 (43.3–138.0)	76%	19%	5%		0.9%		0.0%		9.4%	
	−1	376	277.0 (181.8–377.0)	102.0 (55.0–138.0)	75%	16%	8%		0.5%		0.5%		4.8%	
	0	149	230.0 (155.0–349.0)	93.0 (52.3–130.8)	68%	27%	5%		0.7%		2.7%		1.3%	
7	−3	82	230.5 (141.3–359.3)	75.5 (41.0–114.5)	67%	16%	17%	0.001	0.0%	0.977	0.0%	0.621	8.5%	0.841
	−2	285	247.0 (157.0–351.0)	98.0 (45.3–139.8)	75%	16%	8%		0.4%		0.0%		6.3%	
	−1	314	227.5 (151.0–329.8)	99.0 (44.0–144.0)	69%	23%	8%		0.6%		0.6%		6.1%	
	0	168	233.5 (172.8–311.3)	91.5 (53.3–143.5)	79%	20%	1%		0.6%		0.0%		4.2%	
8	−3	59	215.0 (121.5–303.0)	84.0 (28.0–168.0)	73%	17%	10%	0.268	0.0%	0.957	0.0%	0.735	8.5%	0.276
	−2	223	172.0 (99.0–253.0)	74.0 (34.0–119.0)	78%	20%	3%		0.9%		0.9%		9.4%	
	−1	303	185.0 (121.0–268.5)	88.0 (41.0–146.3)	73%	23%	4%		0.3%		0.3%		5.9%	
	0	286	180.5 (111.3–263.0)	86.0 (47.0–150.0)	77%	17%	6%		0.7%		0.0%		3.8%	
	1	51	161.0 (88.0–221.0)	82.0 (49.0–137.0)	76%	24%	0%		0.0%		0.0%		7.8%	
9	−3	61	202.0 (120.0–256.0)	93.0 (37.5–136.8)	77%	16%	7%	0.053	3.3%	0.144	1.6%	0.735	8.2%	0.096
	−2	165	161.0 (100.8–218.5)	84.0 (47.0–141.5)	75%	19%	5%		0.0%		0.0%		9.1%	
	−1	367	145.5 (86.0–233.0)	77.5 (42.0–137.0)	75%	20%	4%		1.1%		0.3%		7.6%	
	0	518	133.0 (80.0–204.0)	81.0 (42.0–128.5)	82%	16%	2%		0.4%		0.4%		4.6%	
	1	234	120.0 (78.0–191.0)	80.0 (44.0–129.0)	85%	14%	2%		0.4%		0.4%		3.8%	
	2	29	96.0 (47.0–138.0)	51.0 (24.8–89.0)	90%	10%	0%		0.0%		0.0%		0.0%	
10	−1	106	80.5 (43.5–141.3)	80.5 (43.5–141.3)	71%	25%	4%	<0.001	0.0%	0.558	0.0%	0.952	5.7%	0.634
	0	355	83.5 (46.3–140.8)	83.5 (46.3–140.8)	85%	14%	1%		0.8%		0.8%		4.8%	
	1	423	71.0 (36.8–125.0)	71.0 (36.8–125.0)	85%	14%	1%		0.0%		0.9%		4.5%	
	2	265	42.0 (21.0–84.0)	42.0 (21.0–84.0)	88%	12%	0%		0.4%		0.8%		3.8%	
	3	41	20.0 (14.0–50.0)	20.0 (14.0–50.0)	93%	7%	0%		0.0%		0.0%		0.0%	

Data are presented as *n*, % or median (Inter-Quartile Range). *P*-values represent comparisons for each outcome according to the dilation and station at the time of Artificial Rupture of Membranes (AROM), analyzed using the Chi-Square test. NVD, Normal vaginal delivery; AVD, Assisted vaginal delivery; CD, Cesarean delivery; NICU, neonatal intensive care unit, PPH, postpartum hemorrhage. Heat map showing data rates: red for high rates, blue for low.

In multiparas at 4 cm dilation (Figure 5), the AROM to delivery interval decreased with advanced FHS, while CD rates declined significantly. Trends in other outcomes varied across FHS levels, with decreasing rates of low 5-min Apgar scores, NICU admissions, and postpartum hemorrhage observed. The median AROM to delivery interval at 4 cm dilation in multiparas was 252 min (IQR 151-374), 225 min (IQR 135–350), 191 min (IQR 110–307) and 163 min (IQR 90–250) for FHS of −3, −2, −1 and 0, respectively (Figure 5A). When evaluating the outcomes (Figure 5B), CD rates declined significantly from 4% to at FHS −3 to 2% for FHS −2 and −1, and then increased to 3% at FHS of 0 (*p* = 0.002). Low ( ≤ 7) 5-min Apgar score rates decreased gradually from 0.8% to 0% (*p* = 0.182), NICU admission rates decreased from 0.7% at FHS of −3 to 0.4% at FHS of −1 and the increased to 1.3% at FHS 0 (*p* = 0.087), and PPH rates decreased from 5.7% to 2.6% for FHS −3 vs. FHS 0 (*p* = 0.069).

Table 3 depicts a full heat-map table for each cervical dilation in multiparas.

**Table 3:** Heatmap of delivery outcomes and timing from AROM to delivery, stratified by cervical dilation and FHS in multiparas.

**Timing and length of delivery**					**Mode of delivery**				**Outcomes**					
**Dilation**	**Head station**	***n***	**Time AROM to delivery**	**Second stage length**	**NVD**	**AVD**	**CD**	***p*** **-value**	**Apgar 5 min** ≤ **7**	***p*** **-value**	**NICU**	***p*** **-value**	**PPH**	***p*** **-value**
3	−3	794	341.0 (205.0–536.0)	10.0 (5.0–25.0)	84%	7%	9%	0.032	1.4%	0.202	1.1%	0.899	5.7%	0.045
	−2	1181	304.0 (188.0–467.0)	15.0 (7.0–35.0)	89%	5%	6%		0.4%		1.0%		3.0%	
	−1	311	258.0 (153.0–413.0)	19.0 (10.0–49.0)	89%	5%	7%		1.0%		0.6%		3.2%	
	0	49	293.0 (164.0–443.0)	23.0 (11.5–66.0)	84%	12%	4%		0.0%		0.0%		2.0%	
4	−3	2344	252.0 (151.0–374.0)	10.0 (5.0–19.0)	92%	4%	4%	0.002	0.8%	0.182	0.7%	0.870	5.7%	0.069
	−2	2018	225.0 (135.0–350.0)	11.0 (6.0–27.0)	92%	5%	4%		0.4%		0.5%		4.1%	
	−1	671	191.0 (110.0–307.5)	16.0 (10.0–37.0)	92%	6%	2%		0.1%		0.4%		3.9%	
	0	78	163.0 (90.0–249.8)	22.0 (8.0–61.5)	87%	10%	3%		0.0%		1.3%		2.6%	
5	−3	2125	156.0 (88.0–251.0)	10.0 (5.0–21.0)	93%	4%	3%	0.731	0.6%	0.292	0.2%	0.570	6.4%	<0.001
	−2	2186	147.0 (80.0–241.0)	11.0 (6.0–25.0)	94%	4%	2%		0.5%		0.0%		4.2%	
	−1	957	129.0 (74.0–220.0)	15.0 (8.0–32.0)	93%	5%	2%		0.0%		0.3%		3.2%	
	0	167	109.0 (61.0–186.0)	14.0 (7.0–35.0)	93%	5%	2%		0.0%		0.0%		0.6%	
6	−3	1149	99.0 (55.0–179.3)	10.0 (5.0–20.0)	94%	4%	2%	0.398	0.1%	0.796	0.0%	0.155	6.8%	0.003
	−2	1587	101.0 (53.0–178.8)	11.0 (5.0–23.0)	95%	4%	1%		0.2%		0.2%		4.3%	
	−1	862	91.0 (43.0–160.0)	13.0 (6.0–28.3)	96%	3%	1%		0.0%		0.1%		3.4%	
	0	271	72.0 (36.0–137.5)	13.0 (6.0–31.0)	94%	4%	1%		0.0%		0.7%		2.6%	
7	−3	661	79.0 (42.0–135.0)	10.0 (5.0–20.0)	93%	4%	3%	0.223	0.2%	0.929	0.3%	0.022	5.0%	0.221
	−2	1256	68.0 (35.5–128.0)	10.0 (5.0–21.8)	95%	4%	1%		0.3%		0.0%		4.5%	
	−1	884	63.0 (33.0–119.0)	13.0 (7.0–28.0)	95%	3%	1%		0.3%		0.6%		3.3%	
	0	387	51.0 (25.0–95.0)	12.0 (6.0–26.3)	95%	4%	1%		0.0%		0.0%		2.1%	
	1	46	34.0 (13.3–66.5)	7.5 (6.0–10.0)	96%	4%	0%		0.0%		2.2%		2.2%	
8	−3	569	60.0 (31.0–107.0)	10.0 (5.0–21.0)	96%	2%	1%	0.151	0.2%	0.921	0.5%	0.176	6.9%	0.017
	−2	1159	46.0 (23.0–90.8)	10.0 (5.0–20.0)	96%	3%	1%		0.3%		0.1%		3.7%	
	−1	1118	40.0 (20.0–83.0)	10.0 (6.0–21.0)	95%	4%	1%		0.1%		0.1%		3.6%	
	0	727	33.5 (17.0–67.0)	11.0 (5.0–24.0)	97%	2%	1%		0.3%		0.6%		3.3%	
	1	125	27.0 (13.0–51.0)	10.0 (5.5–15.8)	99%	1%	0%		0.0%		0.8%		4.0%	
9	−3	294	42.5 (20.0–82.8)	10.0 (6.0–20.8)	95%	2%	2%	0.006	0.0%	0.888	0.0%	0.281	7.1%	0.066
	−2	700	33.0 (18.0–64.0)	11.0 (5.0–25.0)	95%	3%	1%		0.1%		0.0%		5.4%	
	−1	1038	28.0 (15.0–55.0)	10.0 (5.0–25.0)	96%	3%	1%		0.3%		0.6%		4.8%	
	0	1176	25.0 (13.0–49.0)	11.0 (5.0–24.0)	97%	3%	0%		0.1%		0.3%		3.6%	
	1	348	18.0 (10.0–32.8)	9.0 (5.0–19.0)	98%	1%	1%		0.3%		0.0%		2.9%	
	2	50	16.5 (10.0–27.3)	7.0 (3.5–15.5)	98%	2%	0%		0.0%		0.0%		2.0%	
10	−3	128	17.0 (10.0–33.3)	17.0 (10.0–33.3)	96%	4%	0%	<0.001	0.8%	0.179	0.0%	0.021	5.5%	<0.001
	−2	222	14.0 (8.0–30.0)	14.0 (8.0–30.0)	97%	3%	0%		0.9%		0.9%		7.7%	
	−1	607	16.0 (9.0–32.0)	16.0 (9.0–32.0)	95%	4%	1%		0.2%		0.5%		5.4%	
	0	1725	13.0 (7.0–24.0)	13.0 (7.0–24.0)	97%	2%	0%		0.2%		0.1%		3.4%	
	1	1497	10.0 (5.0–18.0)	10.0 (5.0–18.0)	98%	1%	0%		0.1%		0.1%		3.1%	
	2	798	7.0 (5.0–12.0)	7.0 (5.0–12.0)	99%	1%	0%		0.5%		0.9%		2.1%	
	3	158	5.0 (2.0–10.0)	5.0 (2.0–10.0)	100%	0%	0%		0.0%		0.6%		1.3%	

Data are presented as *n*, % or median (Inter-Quartile Range). *P*-values represent comparisons for each outcome according to the dilation and station at the time of artificial rupture of membranes (AROM), analyzed using the Chi-Square test. NVD, normal vaginal delivery; AVD, assisted vaginal delivery; CD, cesarean delivery; NICU, neonatal Intensive care unit, PPH, postpartum hemorrhage. Heat map showing data rates: red for high rates, blue for low.

## Discussion

This large-scale study shows that duration of labor following AROM at each cervical dilation is influenced by FHS. As the FHS advances within each cervical dilation at time of AROM sub-cohort, the shorter the procedure to delivery duration. This association was stronger in multiparas in comparison to primiparas, perhaps due to the smaller sample size of this sub-cohort. Rates of CDs, low 5-min Apgar scores, NICU admissions, and PPH were also associated with FHS advancement during AROM, indicating that lower fetal head stations corresponded to lower rates of these outcomes, although this did not always reach statistical significance.

As shown previously (25), cervical dilation alone is insufficient in determining length of labor, and combining head station to cervical dilation gives a clearer picture to the progress of labor. The progress of FHS may be influenced by factors such as the fit of the maternal cervix and fetal head circumference, the latter of which has been positively correlated with prolonged second stage and unfavorable maternal and neonatal outcomes (26). Therefore, discussion regarding the optimal timing for AROM during labor induction should include FHS in combination with cervical dilation. It is possible that the one-dimensional approach toward AROM—only including cervical dilation—contributes to the inconsistency of results from the different clinical trials.

The main strength of our study is the large scale of the cohort. This allows for in-depth analysis of each cervical dilation group as well as different subgroups (primiparas vs. multiparas). The fact that results were uniform in most subgroups contributes to the robustness of this study. In the more advanced dilation groups (6–10 cm), numbers were insufficient to draw firm conclusions, however, clinical dilemma regarding AROM at that stage of labor is rare.

The retrospective nature of this study prevented us from ascertaining the optimal timing for AROM, as did the fact that other factors contributing to labor duration, such as administration of oxytocin, were not included in this study. Another potential limitation of this study is confounding by indication: in some cases, AROM may have been performed earlier at higher head stations due to concern for slower labor progress. This underlying factor, rather than AROM timing alone, could partly explain observed differences. While this cannot be excluded in a retrospective design, the consistency of associations across subgroups supports the robustness of our findings. Ultimately, prospective randomized trials will be needed to fully address this question.

Furthermore, this single center study might reflect local practice habits and therefore should be repeated in other delivery units and in different patient populations. As discussed above, clinical trials regarding early vs. late AROM using more complete cervical data are needed before firm recommendations can be made.

We included parturients whose labors culminated in second stage cesarean deliveries in the time-to-delivery analysis, which might affect the timing of delivery of these parturients. In order to examine a cohort of deliveries that were not truncated by CD, a sensitivity analysis excluding CDs was performed, with similar results to the full cohort (Figures 3, 4).

Data collection ended in 2015 because this was the last year for which complete and uniform information was available in our institutional database. While more recent years were not included, the physiological relationships between cervical dilation, fetal head station, and AROM timing are unlikely to be time-dependent, and the clinical implications of incorporating fetal head station into decision-making remain applicable.

These findings concur with previous studies showing the relation between FHS and duration of labor (22–24) as well as studies showing that FHS at the beginning of the second stage of labor is an important determinant of labor outcomes (19–21). However, limited focus has been given to the role of FHS at AROM and its relation to labor outcomes. In previous studies, in which machine learning models were developed to predict obstetrical outcomes, FHS was an important feature for predicting emergency CD (27), severe adverse neonatal outcomes (28), and more (29).

Although previous studies showed FHS estimation to be imprecise (30), the consistency of results throughout each cervical dilation as well as consistency of results amongst different subgroups suggest that perhaps FHS estimation is more accurate than previously thought. Furthermore, a previous study demonstrated that although some variation exists in FHS measurement amongst different medical centers, within the same institution it correlated strongly with labor outcomes (31).

We propose that future research should focus on creating a score combining all cervical features and determining optimal timing for AROM.

## Conclusions

In conclusion, our study emphasizes the significant influence of FHS and cervical dilation on the duration of labor following AROM, particularly in multiparas. Consideration of FHS in AROM timing is crucial, especially for multiparous women, given its impact on various obstetrical outcomes. Further research is essential to fully elucidate the roles of cervical dilation and FHS on the effect of AROM on labor progression, highlighting the need for comprehensive assessments in future clinical trials.

## Data Availability

The data analyzed in this study is subject to the following licenses/restrictions: This is a retrospective review of clinically collected data in patient electronic medical records, therefore protected by privacy laws and not publically available. Requests to access these datasets should be directed to michal.lipschuetz@gmail.com.

## References

[1] De Vivo V Carbone L Saccone G Magoga G De Vivo G Locci M. Early AROM after cervical ripening for induction of labor: a systematic review and meta-analysis of randomized controlled trials. Am J Obstet Gynecol. (2020) 222:320–9. 10.1016/j.ajog.2019.07.049 31398311

[2] Macones GA Cahill A Stamilio DM Odibo AO. The efficacy of early AROM in nulliparous labor induction: a randomized controlled trial. Am J Obstet Gynecol. (2012) 207:403.e1–5. 10.1016/j.ajog.2012.08.032 22959833

[3] Bostanci E Eser A Yayla Abide C Kilicci C Kucukbas M. Early AROM after dinoprostone insert used for the induction of labor: a randomized clinical trial. J Matern Fetal Neonatal Med. (2018) 31:352–6. 10.1080/14767058.2017.1285893 28110590

[4] Makarem MH Zahran KM Abdellah MS Karen MA. Early AROM after vaginal misoprostol for induction of labor: a randomized clinical trial. Arch Gynecol Obstet. (2013) 288:261–5. 10.1007/s00404-013-2747-6 23430026

[5] Levy R Ferber A Ben-Arie A Paz B Hazan Y Blickstein I. A randomised comparison of early versus late AROM following cervical ripening with a Foley catheter. BJOG. (2002) 109:168–72. 10.1111/j.1471-0528.2002.01137.x 11888099

[6] O'Driscoll K Jackson RJ Gallagher JT. Prevention of prolonged labour. Br Med J. (1969) 2:477–80. 10.1136/bmj.2.5655.477 5771578 PMC1983378

[7] Goffinet F Fraser W Marcoux S Breart G Moutquin JM Daris M. Early AROM increases the frequency of fetal heart rate abnormalities. AROM study group. Br J Obstet Gynaecol. (1997) 104:548–53. 10.1111/j.1471-0528.1997.tb11530.x 9166195

[8] ACOG Committee Opinion No. 766 Summary. Approaches to limit intervention during labor and birth. Obstet Gynecol. (2019) 133:406–8. 10.1097/AOG.0000000000003081 30681540

[9] Delgado Nunes V Gholitabar M Sims JM Bewley S Guideline development group. Intrapartum care of healthy women and their babies: summary of updated NICE guidance. BMJ. (2014) 349:g6886. 10.1136/bmj.g6886 25472418 PMC4707719

[10] World Health Organization. WHO Recommendations for Augmentation of Labour. Geneva: World Health Organization. (2014). 25506951

[11] Sheiner E Segal D Shoham-Vardi I Ben-Tov J Katz M Mazor M. The impact of early AROM on mode of delivery and pregnancy outcome. Arch Gynecol Obstet. (2000) 264:63–7. 10.1007/s004040000071 11045324

[12] Segal D Sheiner E Yohai D Shoham-Vardi I Katz M. Early AROM – high risk factor for cesarean section. Eur J Obstet Gynecol Reprod Biol. (1999) 86:145–9. 10.1016/S0301-2115(99)00058-5 10509782

[13] Ghafarzadeh M Moeininasab S Namdari M. Effect of early AROM on dystocia risk and cesarean delivery in nulliparous women: a randomized clinical trial. Arch Gynecol Obstet. (2015) 292:321–5. 10.1007/s00404-015-3645-x 25666481

[14] Smyth RM Markham C Dowswell T. AROM for shortening spontaneous labour. Cochrane Database Syst Rev. (2013) 2013:CD006167. 10.1002/14651858.CD006167.pub4 23780653 PMC11299146

[15] Khodke KS Vijay N. Artificial rupture of membranes and spontaneous rupture of membranes: a systematic review of feto-maternal outcomes. Cureus. (2025) 17:e77760. 10.7759/cureus.77760 39981477 PMC11840793

[16] Khunpradit S Kamkrea S Srisuwan T Mooncheep T. Amniotomy versus expectant management during the active phase of labor defined by the new WHO definition on the duration of labor: a randomized controlled trial. Int J Gynaecol Obstet. (2024) 165:368–74. 10.1002/ijgo.15399 38299786

[17] Bishop EH. Pelvic scoring for elective induction. Obstet Gynecol. (1964) 24:266–8. 14199536

[18] Gomez Slagle HB Fonge YN Caplan R Pfeuti CK Sciscione AC Hoffman MK. Early vs expectant artificial rupture of membranes following Foley catheter ripening: a randomized controlled trial. Am J Obstet Gynecol. (2022) 226:724.e1–e9. 10.1016/j.ajog.2021.11.1368 35135684

[19] Ashwal E Fan IY Berger H Livne MY Hiersch L Aviram A. The association between fetal head station at the first diagnosis of the second stage of labor and delivery outcomes. Am J Obstet Gynecol. (2021) 224:306.e1–e6. 10.1016/j.ajog.2020.09.006 32926858

[20] Ludvigsen E Skjeldestad FE. Station of the fetal head at complete cervical dilation impacts duration of second stage of labor. Eur J Obstet Gynecol Reprod Biol X. (2020) 7:100100. 10.1016/j.eurox.2019.100100 32715290 PMC7379142

[21] Hochler H Guedalia J Lipschuetz M Walfisch A Yagel S Guedalia Friedman E. Normal labor curve in twin gestation. Am J Obstet Gynecol. (2021) 225:546.e1–11. 10.1016/j.ajog.2021.07.019 34363782

[22] Haberman S Atallah F Nizard J Buhule O Albert P Gonen R. A novel partogram for stages 1 and 2 of labor based on fetal head station measured by ultrasound: a prospective multicenter cohort study. Am J Perinatol. (2021) 38:e14–20. 10.1055/s-0040-1702989 32120420

[23] Hamilton EF Simoneau G Ciampi A Warrick P Collins K Smith S. Descent of the fetal head (station) during the first stage of labor. Am J Obstet Gynecol. (2016) 214:360.e1–6. 10.1016/j.ajog.2015.10.005 26475422

[24] Hamilton EF Warrick PA Collins K Smith S Garite TJ. Assessing first-stage labor progression and its relationship to complications. Am J Obstet Gynecol. (2016) 214:358.e1–8. 10.1016/j.ajog.2015.10.016 26478103

[25] Barnea O Luria O Jaffa A Stark M Fox HE Farine D. Relations between fetal head descent and cervical dilatation during individual uterine contractions in the active stage of labor. J Obstet Gynaecol Res. (2009) 35:654–9. 10.1111/j.1447-0756.2008.00996.x 19751323

[26] Lipschuetz M Cohen SM Israel A Baron J Porat S Valsky DV. Sonographic large fetal head circumference and risk of cesarean delivery. Am J Obstet Gynecol. (2018) 218:339.e1–7. 10.1016/j.ajog.2017.12.230 29305249

[27] Guedalia J Lipschuetz M Novoselsky-Persky M Cohen SM Rottenstreich A Levin G. Real-time data analysis using a machine learning model significantly improves prediction of successful vaginal deliveries. Am J Obstet Gynecol. (2020) 223:437.e1–15. 10.1016/j.ajog.2020.05.025 32434000

[28] Guedalia J Sompolinsky Y Novoselsky Persky M Cohen SM Kabiri D Yagel S. Prediction of severe adverse neonatal outcomes at the second stage of labour using machine learning: a retrospective cohort study. BJOG. (2021) 128:1824–32. 10.1111/1471-0528.16700 33713380

[29] Lipschuetz M Guedalia J Rottenstreich A Novoselsky Persky M Cohen SM. Prediction of vaginal birth after cesarean deliveries using machine learning. Am J Obstet Gynecol. (2020) 222:613.e1–12. 10.1016/j.ajog.2019.12.267 32007491

[30] Buchmann E Libhaber E. Interobserver agreement in intrapartum estimation of fetal head station. Int J Gynaecol Obstet. (2008) 101:285–9. 10.1016/j.ijgo.2007.11.020 18222452

[31] Guedalia J Lipschuetz M Cohen SM Sompolinsky Y Walfisch A Sheiner E. Transporting an artificial intelligence model to predict emergency cesarean delivery: overcoming challenges posed by interfacility variation. J Med Internet Res. (2021) 23:12–e28120. 10.2196/28120 34890352 PMC8709908

